# Assessment of Healthcare Professionals' and Students' Perspectives and Intentions for Raising Public Awareness and Comprehension of Antimicrobial Resistance

**DOI:** 10.1155/cjid/4106594

**Published:** 2025-09-15

**Authors:** Malik Suliman Mohamed, Elkhanssa Abdelhameed Ahmed Elhag, Alnada Ibrahim, Mona Timan Idriss, Eyman Mohamed Eltayib, Tilal Elsaman, Magdi Awadalla Mohamed

**Affiliations:** ^1^Department of Pharmaceutics, College of Pharmacy, Jouf University, Sakaka 72388, Saudi Arabia; ^2^Department of Pharmacy Practice, Faculty of Pharmacy, University of Khartoum, Khartoum 11111, Sudan; ^3^Department of Pharmaceutics, Faculty of Pharmacy, University of Khartoum, Khartoum 11111, Sudan; ^4^Department of Medical Sciences and Preparation Year, Northern College of Nursing, Arar 73312, Saudi Arabia; ^5^Department of Pharmaceutical Chemistry, College of Pharmacy, Jouf University, Sakaka 72388, Saudi Arabia

**Keywords:** antimicrobial resistance, awareness, healthcare students, professionals, public, theory of planned behavior

## Abstract

**Background:** Limited public knowledge of antimicrobial agents contributes to their misuse and antimicrobial resistance (AMR).

**Objective:** This study aimed to assess the intentions of healthcare students and professionals in promoting public awareness and understanding of AMR.

**Methods:** A 31-item survey based on the theory of planned behavior (TPB) and the health belief model was developed, incorporating intention, attitudes, subjective norms, perceived behavioral control, and perceived benefits. The survey aligned with global and national AMR action plans and was distributed among healthcare professionals and students in Al-Jouf, Saudi Arabia, via Google Forms. Responses were analyzed using frequencies, percentages, correlations between constructs, and ordinal logistic regression to assess significant associations between intention and other variables.

**Results:** A total of 572 participants completed the survey, comprising 59.4% males and 40.6% females. Over one-third were younger individuals, and about one-third were undergraduate students or had less than 5 years of experience. Most respondents (74%) expressed an intention to educate the public on AMR, with a median intention score of 24 (out of 30). Intention showed strong positive correlations with past behavior (*r* = 0.703), subjective norms (*r* = 0.695), perceived behavioral control (*r* = 0.690), and perceived benefits (*r* = 0.683), while attitudes had a weak correlation (*r* = 0.122). Attitudes also had low correlations with other constructs (*r* = 0.137–0.278). Among predictors, subjective norms significantly influenced intention (*p* < 0.001), while other factors showed no significant predictive relationship.

**Conclusion:** Healthcare students and professionals exhibited a strong inclination toward educating the public on responsible antimicrobial use and AMR. The findings underscore the complex interplay of factors influencing this intention, with subjective norms playing a key role, highlighting the impact of social pressure. Identifying these contributing factors can inform targeted strategies for healthcare professionals and students, enabling broader educational outreach and strengthening AMR control efforts.

## 1. Introduction

Microbial infections pose a significant threat to human health and remain a leading cause of morbidity and mortality worldwide. Although the discovery of antimicrobial agents has led to major advancements in controlling these infections, the emergence of antimicrobial resistance (AMR) has become a growing concern [1]. The widespread use of antimicrobials and the increasing rates of resistance are critical global health challenges due to their profound impact on public health. Studies indicate that resistance rates are generally lower in high-income countries compared to middle- and low-income nations [2].

The rational use of antimicrobials involves selecting and administering these drugs based on clinical necessity, scientific evidence, and cost-effectiveness. However, the irrational use of antimicrobials remains one of the primary drivers of AMR [3]. Several factors contribute to the development of resistance, including human activities, animal-related practices, and environmental influences. Self-medication, the availability of substandard or counterfeit drugs, antibiotic-containing cosmetics, low vaccination coverage, and the use of antimicrobials in animal husbandry, food production, and aquaculture are all associated with the acceleration of AMR [4].

Healthcare providers play a crucial role in the rational prescription of antimicrobials, but their knowledge and prescribing behaviors have been evaluated for their potential contribution to AMR. While many healthcare professionals demonstrate confidence in diagnosing infectious diseases and selecting appropriate treatments, gaps in knowledge regarding AMR and rational prescribing practices have been identified [5]. These gaps can impact their ability to educate patients and the broader community on the risks of AMR. Factors influencing antibiotic prescription among healthcare professionals include time constraints, adherence to clinical guidelines, peer prescribing habits, pharmaceutical marketing pressures, and societal expectations [6]. Such factors can significantly impact their role in promoting public awareness about AMR. On the other hand, healthcare providers who receive adequate AMR education during their undergraduate studies or through professional training and workshops are less likely to prescribe antibiotics inappropriately [7]. Similarly, individuals involved in antimicrobial use, such as those in the agriculture and animal health sectors, who adhere to antimicrobial stewardship policies, contribute significantly to combating AMR. The implementation of antimicrobial stewardship programs serves as a cornerstone in strengthening healthcare systems and addressing the global AMR crisis [8].

Various barriers, including time constraints, language differences, socioeconomic factors, healthcare system limitations, cultural influences, and work-related stress, can significantly hinder efforts to enhance public health literacy. However, healthcare providers play a crucial role in patient education by fostering true partnerships with shared authority and responsibility. Through this collaborative approach, they actively contribute to AMR [9]. Research has shown that patient education and health literacy interventions significantly improve self-efficacy and adherence to treatment, ultimately leading to better health outcomes [10]. A study utilizing one-on-one interviews, based on the theory of planned behavior (TPB), assessed clinicians' knowledge, attitudes, and the necessary interventions to minimize unnecessary antibiotic prescriptions for acute respiratory infections. While clinicians expressed the intention to prescribe antibiotics appropriately, the study identified several barriers, including the need for enhanced patient education, which were recommended for targeted intervention [11].

Expanding the scope of patient education through the involvement of medical students, preferably under supervision, has also proven to be beneficial. Research suggests that student-led education initiatives improve patients' health knowledge, disease management, and medication adherence while also benefiting students by enhancing their communication skills, learning effectiveness, and overall quality of patient education [12]. Medical students can play a valuable role in healthcare delivery, extending their contributions to population health management and public education [13]. Evidence supports the effectiveness of student-led educational interventions, demonstrating improvements in key health indicators such as glycosylated hemoglobin A1C, low-density lipoprotein (LDL), and blood pressure levels among diabetic patients [14]. Similarly, participation in sexual health peer education programs has been shown to enhance both medical students' and secondary school pupils' knowledge on the topic [15]. Given these findings, assessing healthcare students' willingness and preparedness to raise public awareness about AMR could be instrumental in aligning with the strategic objectives of the National Action Plan on AMR. Strengthening communication, education, and training among future healthcare professionals will be essential in addressing this global health challenge.

The TPB is widely utilized in designing and evaluating behavior change interventions. As a well-established theoretical framework in social psychology, TPB has been extensively validated and recognized for its effectiveness in predicting intentions and health-related behaviors [16]. According to the TPB model, behavioral intentions are influenced by key factors, including attitudes, subjective norms, and perceived behavioral control. Research suggests that interventions based on theoretical frameworks, such as TPB, are generally more effective than those lacking a theoretical foundation. Additionally, TPB considers past behavior as an indirect factor influencing future actions, as prior experiences shape attitudes, subjective norms, and perceived behavioral control, which in turn determine intentions [17]. Numerous studies employing TPB have explored various aspects of antibiotic use, including prescribing behaviors, the dispensing of subtherapeutic antibiotic doses, and barriers to rational prescribing in multiple countries, including China, Egypt, Saudi Arabia, and Qatar [6, 18–21]. Furthermore, a validated TPB-based questionnaire has been used to assess changes in the professional behaviors of medical students [22]. In addition to TPB, growing evidence supports the application of the health belief model (HBM) in understanding health behaviors among both healthcare professionals and the general public. The HBM is one of the most widely used models in health behavior research and includes constructs such as perceived susceptibility, perceived severity, perceived barriers, and perceived benefits. Among these, perceived benefits is particularly relevant when assessing the motivational factors that influence health-promoting behaviors not directly tied to personal health risks [23–25]. In this study, the target behavior involves healthcare professionals' and students' intentions to raise public awareness about AMR, a form of advocacy and communication rather than a personal health action. Therefore, while other HBM constructs were considered, they were deemed less applicable in this context. While the TPB provides a well-established framework for predicting behavioral intentions through constructs such as attitudes, subjective norms, and perceived behavioral control, it does not explicitly address individuals' evaluations of the potential advantages of performing a behavior. To strengthen the predictive power of the model in the context of this study, we incorporated the “perceived benefits” construct from HBM to assess the extent to which participants believe their involvement in raising AMR awareness would have a meaningful impact. This hybrid model offers a more comprehensive understanding of both the intention formation (via TPB) and motivational valuation (via HBM). Perceived benefits are central to health-related decision-making and have been shown in prior research to significantly influence engagement in preventive health behaviors [26]. Since raising awareness about AMR involves proactive communication and public engagement, often requiring additional time and effort from healthcare professionals and students, understanding their perception of the benefits of such actions is critical. Therefore, integrating this construct was intended to provide a more comprehensive explanation of the motivational factors driving their intentions.

Local assessments using the TPB revealed that physicians have insufficient knowledge of antibiotic-resistant bacteria but exhibit a strong behavioral intention to prescribe antibiotics. This is coupled with favorable attitudes and satisfactory social pressure towards antibiotic use [20]. Likely, a study investigating medical students' knowledge of antibiotic use and resistance found that while respondents recognized the rise in infections caused by resistant bacteria, they believed that healthcare workers could effectively reduce antibiotic resistance in Saudi Arabia [27]. In light of this, a comprehensive literature review of reports from Saudi Arabia highlighted a significant research gap regarding the intentions of healthcare students and professionals to raise public awareness and comprehension of AMR. To address this need, the present study was conducted to assess their perspectives and intentions on enhancing public awareness and comprehension of AMR.

## 2. Materials and Methods

### 2.1. Study Design and Settings

This descriptive, cross-sectional survey study was conducted online. A questionnaire, created using Google Forms, was designed and distributed through social media platforms targeting health professionals and students in the Al-Jauf region of Saudi Arabia, from March to June 2023. The Google Form included online informed consent, the research purpose, the ethical approval number, and the researcher's email for any inquiries.

### 2.2. Study Population and Sampling

The study targeted individuals from various health-related disciplines, medicine, pharmacy, dentistry, nursing, physiotherapy, and medical laboratory sciences, within the Al-Jauf province. The participant pool included both licensed healthcare professionals (those currently practicing or employed in clinical or public health settings) and undergraduate students enrolled in health-related academic programs. The inclusion of students is justified by their growing role in public health awareness activities, particularly in Saudi Arabia. Although their participation is not typically mandated by ethical or professional obligations, many students engage in such initiatives through structured extracurricular and volunteer programs within universities. Moreover, national efforts, such as the official platform for health volunteering (https://volunteer.srca.org.sa/), actively encourage student involvement in public health education, including campaigns addressing AMR. Given these factors, students represent an important and relevant segment of future healthcare providers and public health advocates, and their perspectives are valuable for understanding readiness and intention to promote AMR awareness. To improve clarity and accuracy, the term “respondents” or “participants” is used throughout the manuscript to reflect this diverse population. The sample size was estimated using the online form of Cochran's equation (https://calculator.academy/cochrans-sample-size-calculator/) with the formula “*n* = *Z*^2^ × *p* × *q*/*e*^2^”. This formula allows the determination of an appropriate sample size, given different combinations of confidence, precision, and variability. The variables in the equation are: “*e*” is the desired level of precision (margin of error 5% or 0.05), “*P*” is the population proportion expected to respond 50%, “*q*” is 1 − *p*, and *Z* value corresponding to the desired confidence level (1.96 for 95% confidence). Based on the calculation results, the sample was estimated to be 377; however, the study aimed at collecting more responses to increase the validity of the findings.

### 2.3. Survey Instrument Construction and Validation

Data were collected using a structured questionnaire. The questionnaire was developed taking into consideration the TPB framework which suggests attitudes, subjective norms, and perceived behavioral control as factors that can influence behavioral intentions. Moreover, the TPB considers past behavior to have an indirect influence on future actions. Guided by the HBM, the questionnaire explored the impact of perceived benefits on behavioral intentions. This model focuses on individuals' perceptions of health threats and their decision-making processes based on the value they place on a specific goal and the likelihood that their actions will achieve that goal. The combination of the TPB and HBM allows the questionnaire to consider both social and cognitive factors that influence behavior.

The questionnaire consisted of seven domains covering (1) sociodemographic characteristics (six items); (2) intention to raise the public awareness and understanding of AMR (six items); (3) attitude toward raising the public awareness and understanding of AMR (six items: three positive and three negative statements); (4) subjective norms or social pressure (three items); (5) perceived behavioral control (three items); (6) past behavior “participated in raising the public awareness” (three items); and (7) perceived benefits (four items).

The questionnaire was developed in alignment with the key strategies and initiatives outlined in both the Global Action Plan and the Saudi Arabian National Action Plan for combating AMR [28, 29]. It covers essential topics such as infection control, responsible antibiotic use, and public awareness. This study is specifically based on Strategic Objective 1 of the National Action Plan: “Improve awareness and understanding of AMR through effective communication, education, and training”.

The questionnaire was initially developed in English and later translated into Arabic, followed by a back-translation into English by independent experts to ensure consistency. Before data collection commenced, the questionnaire underwent a validation process to ensure clarity, relevance, and content accuracy. A panel of experts, comprising a microbiologist, a clinical pharmacist, and a biostatistician, all affiliated with health-related academic institutions, reviewed the instrument. Their combined expertise contributed to ensuring that the questionnaire was scientifically sound, contextually appropriate, and aligned with the study's objectives. To assess the internal consistency of the questionnaire constructs, Cronbach's alpha was calculated for each major domain. The results indicated acceptable to high reliability across most constructs: intention (6 items): *α* = 0.88, attitudes (6 items): *α* = 0.71, subjective norms (3 items): *α* = 0.84, perceived behavioral control (3 items): *α* = 0.81, past behavior (3 items): *α* = 0.79, and perceived benefits (4 items): *α* = 0.83. These values demonstrate that the items within each construct had satisfactory internal consistency, supporting the reliability of the instrument used in this study.

### 2.4. Data Collection

The survey was administered electronically using Google Forms and was made available in both English and Arabic to accommodate participants' language preferences. To maximize reach and participation, the survey link was initially distributed through various social media platforms commonly used by students and healthcare professionals. Data collection continued until the number of responses exceeded the calculated sample size. This approach was taken to ensure that, after data cleaning based on the inclusion and exclusion criteria, the final sample would meet or surpass the required minimum size. Upon closure of the survey, responses were exported to Microsoft Excel for data cleaning and preliminary coding. Responses that did not meet the inclusion criteria were excluded. Numerical codes were assigned to the relevant variables, and the cleaned dataset was then imported into SPSS for statistical analysis.

### 2.5. Measurements

The primary outcome of the study was the “intention to raise public awareness and understanding of AMR,” which was evaluated using a 5-point Likert scale ranging from 1 (*Strongly Disagree*) to 5 (*Strongly Agree*). For the overall assessment of intention, responses were dichotomized as follows: participants who selected “*Strongly Agree*” or “*Agree*” were classified as having an intention to raise public awareness, whereas those who responded with “*Neutral*,” “*Disagree*,” or “*Strongly Disagree*” were categorized as not intending to do so. This dichotomization was based on theoretical and practical considerations. Behaviorally, the “Neutral” response does not convey a clear commitment to act and is thus more aligned with non-intention than intention. In line with the TPB, intention implies a conscious plan or decision to perform a behavior. Uncertainty, reflected in neutral responses, indicates a lack of that plan. Combining “No” and “Uncertain” responses helped distinguish between respondents who had a definitive intention and those who either rejected or had not yet formed an intention to act. Methodologically, combining these categories also enhanced the statistical robustness of regression analyses by avoiding sparse data in separate outcome categories.

Several potential factors were hypothesized to influence this intention, including sociodemographic characteristics, attitude, subjective norms, perceived behavioral control, past behavior, and perceived benefits. The sociodemographic variables examined in the study included gender, age, marital status, years of experience, field of education, and education level. Attitudes, subjective norms, perceived behavioral control, past behavior, and perceived benefits were measured using a 5-point Likert scale (1 = *Strongly Disagree* to 5 = *Strongly Agree*). Attitudes were assessed through three positive and three negative statements, with the negative statements reverse-coded during data analysis. To categorize participants, attitude responses were dichotomized: those who answered “*Strongly Agree*” or “*Agree*” were classified as having a positive attitude, while those who responded with “*Neutral*,” “*Disagree*,” or “*Strongly Disagree*” were categorized as having a negative attitude.

### 2.6. Data Analysis

Data analysis was conducted using the Statistical Package for Social Sciences version 25 (SPSS Inc., Chicago, IL, USA). The collected data were cleaned and anonymously encoded into Excel sheets before being exported to SPSS for analysis. Descriptive statistics were used to summarize the study variables, including frequencies, proportions, medians, and interquartile ranges (IQRs), depending on the normality of data distribution. Spearman's rho correlation coefficient was applied to assess the strength and direction of relationships between intention, attitudes, subjective norms, perceived behavioral control, past behavior, and perceived benefits. The interpretation of correlation coefficient values was as follows: very strong correlation (±0.90 to ±1.00); strong correlation (±0.70 to ±0.89); moderate correlation (±0.40 and ± 0.69); weak correlation (±0.10 and ± 0.39); and negligible correlation (< ±0.10) [30]. The correlation coefficients were considered significant when *p* value is less than 0.05.

Binary logistic regression analysis was employed to identify predictors of healthcare professionals' and students' intention to raise public awareness about AMR. Explanatory variables of the proposed model included the sociodemographic characteristics and the cognitive variables (attitudes, subjective norms, perceived behavioral control, past behavior, and perceived benefit). Multicollinearity was checked before including independent variables with strong correlation in the regression model. Perceived behavioral control, past behavior, and perceived benefit were not included in the binary logistic analysis, as they were strongly correlated with each other based on a cut-off point of ±0.7 as reported in the literature [31]. The crude and adjusted odds ratio in binary logistic regression model with their 95% confidence interval were used to measure the strength and significance of the association between the various independent variables and intention to raise public awareness of AMR.

### 2.7. Ethical Considerations

The Local Committee of Bioethics (LCBE) of Jouf University reviewed and granted an ethical approval with the number: 1-06-44, dated 07 February 2023. The front page of the Google Form included brief information about the project and the researchers, and informed online consent was required from all participants. The participants had the right to continue or withdraw from the study at any time, making participation entirely voluntary. The form did not collect any identifiable information from the participants. The questionnaire was anonymized, and confidentiality of information obtained was maintained.

## 3. Results

### 3.1. Participants' Sociodemographic Characteristics

Five hundred seventy-two participants were recruited in this study, of whom 340 (59.4%) were males. Of the participants, 195 (34.1%) were in the age group 18–24 years, 312 (54.5%) had a bachelor's degree, 227 (39.7%) were from the pharmacy field, 175 (30.6%) had practice experience of less than five years, and 290 (50.7%) were single.  Table 1 provides details of sociodemographic characteristics.

### 3.2. Descriptive Statistics of Intention and Related Constructs Among Healthcare Professionals and Students

The descriptive statistics of the scores of participants' intention toward raising public awareness of antimicrobial use and resistance, as well as the constructs used to assess it, are presented in Table 2. Overall, the median intention score is 24 (with reference to a maximum possible score of 30).

### 3.3. Correlations Between Intention, Attitude, Subjective Norms, Perceived Behavioral Control, Past Behavior, and Perceived Benefits

The correlations between intention and the constructs used to assess it are presented in Table 3. There were moderate to strong correlations between intention and all constructs, except for attitude (*r* = 0.122). The attitude domain also showed weak positive correlations with all other constructs, with *r* ranging from 0.137 to 0.278.

### 3.4. Healthcare Professionals and Students' Responses to Intention Items on Raising Public Awareness of AMR

Of the study participants, 425 (74.3%) expressed their intention to raise public awareness and understanding of AMR. The remaining participants (one-quarter of the total) were either not intending to do so or were uncertain about their decision at the time of the study. For individual intention items, participants' responses ranged from 59.4% to 73.6%. The two items with the highest percentage (73.6%) were “educating the community about personal and environmental hygiene” and “collaborating with other healthcare professionals for infection prevention and control.” Table 4 provides more details on participants' responses to the different intention items.

### 3.5. Predictors of Healthcare Professionals and Students' Intention Toward Raising Public Awareness of AMR

In the overall sample, 425 participants (74.3%) expressed their intention to raise public awareness of antimicrobial use and resistance. The subjective norm score was a significant predictor of participants' intention (*p* < 0.001, adjusted OR 1.823; 95% CI 1.65–2.02) (Table 5). In the unadjusted analysis (crude odds ratio), years of experience appeared as a significant predictor of participants' intention (*p* < 0.043), with a reported lower intention among participants with less than 5 years of experience compared to those with more than 10 years of experience (*p*=0.006, crude OR 0.428; 95% CI 0.233–0.788). However, in the adjusted analysis, years of experience was not a significant predictor for this group (*p*=0.245, adjusted OR 0.513; 95% CI 0.166–1.58). The observed difference in the unadjusted analysis was reduced when other variables were included. A similar pattern was observed with attitudes (positive vs. negative/neutral) and age range. In the unadjusted analysis, attitude was a significant predictor (*p*=0.007, crude OR 1.872; 95% CI 1.19–2.94), whereas the adjusted odds ratio showed no significant difference in participants' intention based on their attitude (*p*=0.295, adjusted OR 1.416; 95% CI 0.738–2.73). There were no significant differences in participants' intention based on gender, education level, field of study, or marital status. Table 5 presents the details of the binary logistic regression analysis.

## 4. Discussion

The current study assessed the perspectives and intentions of healthcare professionals and students in Al-Jauf province, Saudi Arabia regarding their role in raising public awareness and comprehension of AMR. A significant majority of the participants (74%) expressed an intention to educate the public about responsible antimicrobial use and AMR. This high level of intention suggests a strong potential for healthcare professionals and students to play a key role in educating the public about AMR. The finding also offers a promising foundation for developing and implementing public education initiatives, as a large proportion of the participants was receptive to engaging in such activities. Aggregate published reports highlight the positive attitudes and intentions of healthcare professionals and students toward public education concerning responsible antimicrobial use and AMR, while concurrently emphasizing the necessity of formalized educational interventions and resource provision to optimize knowledge acquisition and engagement in AMR-related initiatives [32–34].

The present study demonstrated significant correlations (*p* < 0.01) between participants' intention to raise public awareness about AMR and the studied factors. Specifically, intention demonstrated a strong positive correlation with past behavior (*r* = 0.703), moderate positive correlations with subjective norms (*r* = 0.695), perceived behavioral control (*r* = 0.690), and perceived benefits (*r* = 0.683). The strong correlation of intention with past behavior suggests that prior engagement in similar activities significantly influences participants' current intentions. This highlights the importance of fostering their early and consistent involvement in public health initiatives related to AMR. The moderate correlations with subjective norms, perceived behavioral control, and perceived benefits highlight the considerable impact of social influence, self-efficacy, and perceived advantages in shaping intentions. Participants are more likely to intend to educate the public if they believe that others expect them to do so, if they feel capable of doing it, and if they perceive clear benefits from their actions.

Conversely, a weak positive correlation was observed between intention and attitudes (*r* = 0.122), as well as between attitudes and other constructs (*r* = 0.137–0.278), which merits further consideration. While participants may express generally positive attitudes toward raising awareness about AMR, this does not necessarily translate into strong behavioral intention. Several contextual factors may explain this disconnect. For instance, participants, especially students or early-career professionals, may lack specific knowledge, confidence, or communication skills needed for effective public education. Moreover, external barriers such as time constraints, lack of institutional support, or uncertainty about their role in public health outreach may limit their ability or willingness to act on their attitudes. These findings align with other behavioral intention studies that suggest attitudes, while important, are not always the strongest predictor of intention, particularly in complex or multistakeholder health behavior contexts [35, 36]. It is also possible that the attitude construct, while theoretically valid, may require further contextual refinement in future research to more accurately capture the type of attitudes most predictive of intention in the setting of AMR awareness. Published literature [35–37] has demonstrated the complex interplay between individual attitudes, social influences, perceived risks and benefits, and behavioral intentions in the context of antimicrobial use and resistance. This highlights the importance of understanding these factors to develop effective strategies for promoting responsible antimicrobial use and combating AMR.

Participants' responses to individual intention items revealed varying degrees of willingness to engage in AMR-related education and advocacy. Reported intention levels ranged from 59.4% to 73.6%, with the highest proportions (both at 73.6%) related to educating the public on personal and environmental hygiene, and collaborating with other healthcare professionals for infection prevention and control. Studies have emphasized the critical role of education and interprofessional collaboration in promoting infection prevention and controlling AMR, and they support the notion that targeted educational interventions can enhance intentions and behaviors related to hygiene and collaborative infection control efforts [38, 39]. Collaboration between healthcare professionals in patients' education about infection control and many other health aspects was highlighted in many studies as being of great value to patients' health [40, 41]. Around two-thirds of the surveyed population mentioned that they want to use the social media to raise the public awareness about the antimicrobial's use and misuse. The role of social media as means of education was highlighted in several studies [42, 43]; however, not all the information in social media is reliable and should be watched carefully by healthcare providers. Healthcare professionals' intention to correct health misinformation on social media was studied using the TPB. The study revealed that they prefer public methods, and their intention to correct misinformation was indirectly predicted by their perceived organizational support [44].

To better understand the underlying factors shaping this willingness, we further analyzed key psychosocial and demographic predictors of overall intention. Subjective norm score was a significant predictor for participants' intention. In a parallel finding, subjective norms significantly predicted physicians' intentions to utilize antimicrobial practice guidelines [37]. In the unadjusted analysis (crude odds ratio), years of experience was a significant predictor of participants' intention (*p* < 0.043), with participants who had less than 5 years of experience reporting lower intention compared to those with more than 10 years of experience. However, in the adjusted analysis, years of experience were not a significant predictor of participants' intention. The initial finding, that more experienced healthcare professionals and students are more likely to intend to educate the public about AMR, may be influenced by various factors beyond experience alone. The adjusted analysis, which accounts for these variables, offers a more accurate understanding of the true relationship between experience and the intention to educate the public about AMR. While experience is valuable, research also highlights that supportive organizational structures, such as access to resources and time, play a crucial role in enabling healthcare professionals to engage in educational initiatives. Without such support, even experienced individuals may struggle to prioritize public education on AMR [45]. Similar to experience, attitude showed a discrepancy between the unadjusted and adjusted analyses. Initially, attitude appeared to be significant, but once other variables were considered, it was no longer a predictor. This suggests that while a positive attitude might seem like a logical driver of intention, other factors play a more influential role. A similar pattern was observed with age, where the adjusted analysis found that age did not predict intention. This implies that the desire to educate is not restricted to any specific age group among these professionals and students. These findings emphasize that the intention to educate about AMR is a complex issue, and simple demographic or attitudinal factors may not be strong predictors. They highlight the need for a broader understanding of the underlying drivers of intention [46]. Our findings also revealed that participants' intentions to educate the public about AMR were not significantly influenced by gender, education level, field of study, or marital status, which aligns with existing research [47]. This suggests that these demographic factors may not be strong predictors of the intention to engage in public education on AMR.

It is important to note that, for the purpose of analysis, the outcome variable was dichotomized by grouping “No” and “Uncertain” responses into a single category representing nonintention. This decision was guided by the conceptual framework of the TPB, which defines intention as a clear and affirmative decision to engage in a specific behavior. Since “Uncertain” responses do not reflect a firm behavioral commitment, they were considered more closely aligned with nonintention in this context. This dichotomization also supported the stability of the logistic regression model by ensuring sufficient cell sizes. Nonetheless, we acknowledge that “Uncertain” responses may reflect motivational ambiguity rather than outright rejection. Future research could explore these gradations more precisely using multinomial or ordinal logistic regression models to better capture the spectrum of behavioral readiness among healthcare professionals and students.

Our study has some limitations. Since the survey was distributed online via social media, the perspectives of individuals who do not use these platforms were not captured. Moreover, data were gathered through a self-administered questionnaire rather than interviews, which may affect accuracy. This study did not explore prevalent practices regarding healthcare professionals' educational efforts toward patients and the broader community. Future cohort studies should consider an “annual face-to-face interview approach” to obtain a more representative sample. Furthermore, the findings from this survey have limited generalizability due to factors such as sample size, selection bias, and the fact that all respondents were Internet users. Although the study employed a theory-informed instrument based on the TPB and the HBM, we acknowledge a key limitation regarding psychometric validation. Particularly, while the questionnaire underwent expert review for content and face validity and was pilot-tested to refine clarity and relevance, formal assessment of construct validity (e.g., through exploratory or confirmatory factor analysis) was not conducted. This limits our ability to make strong inferences about the latent structure of the constructs measured. We have noted this limitation and recommend that future research involving the same tool, especially with larger sample size, undertake a comprehensive psychometric validation process, including factor analysis. Despite these constraints, this study is the first to examine the role of healthcare students and professionals in raising public awareness and understanding of AMR within the study area. It highlights key factors associated with a greater intent to promote awareness. Future research should aim to identify additional elements that influence the practices of healthcare professionals and students, enhance educational initiatives, and extend outreach efforts to a wider community to contribute more effectively to AMR control.

## 5. Conclusions

Overall, this study provides valuable insight into the intentions of healthcare professionals and students to engage in public awareness campaigns concerning antimicrobial use and resistance. The strong expression of their intent underscores a potential resource for combating the growing threat of AMR. Notably, the study revealed that subjective norms, the perceived social pressure to perform a behavior, were significant predictors of these intentions. This highlights the importance of social influence and peer support in promoting responsible antimicrobial stewardship. However, it is equally important to acknowledge that despite variations in gender, educational level, and field of education, there were no statistically significant differences in participants' stated intentions. This may suggest a widespread, shared commitment to raising public awareness across diverse demographic and professional groups. Future research should delve deeper into the potential interventions that leverage these social factors to enhance public education efforts. Furthermore, qualitative studies could provide more insights into the experiences and perspectives of participants. Assessing the actual behaviors that result from these intentions would be the logical next step.

## Figures and Tables

**Table 1:** Participants' sociodemographic characteristics (*N* = 572).

Variables	Frequency (%)
*Gender*	
Male	340 (59.4)
Female	232 (40.6)

*Age in years*	
18–24 years	195 (34.1)
25–30 years	145 (25.3)
31–40 years	139 (24.3)
More than 41	93 (16.3)

*Marital status*	
Single	290 (50.7)
Married	241 (42.1)
Divorced	30 (5.2)
Widow	11 (1.9)

*Years of experience*	
Undergraduate student	168 (29.4)
< 5 years	175 (30.6)
5–10 years	123 (21.5)
> 10 years	106 (18.5)

*Education level*	
Undergraduate student	153 (26.7)
Bachelor	312 (54.5)
Master	51 (8.9)
Doctorate (PhD)	56 (9.8)

*Field of education*	
Pharmacy	227 (39.7)
Medicine	91 (15.9)
Nursing	125 (21.9)
Medical laboratory science	64 (11.2)
Physiotherapy	45 (7.9)
Dentistry	20 (3.5)

**Table 2:** Descriptive statistics of intention, attitude, subjective norms, perceived behavioral control, past behavior, and perceived benefits among healthcare professionals and students (*N* = 572).

Construct	Number of items	Score range	Median score (IQR)
Intention	6	6–30	24 (20–27)
Attitudes	6	12–30	19 (18–21)
Subjective norms	3	3–15	12 (9–13)
Perceived behavioral control	3	3–15	12 (9–13)
Past behavior	3	3–15	12 (12–14)
Perceived benefits	4	4–20	16 (12–18)

Abbreviation: IQR = interquartile range.

**Table 3:** Spearman rho's correlations between intention, attitude, subjective norms, perceived behavioral control, past behavior, and perceived benefits (*N* = 572).

	Intention	Attitude	Subjective norms	Perceived behavioral control	Past behavior	Perceived benefits
Intention	1					
Attitude	0.122^∗∗^	1				
Subjective norms	0.695^∗∗^	0.137^∗∗^	1			
Perceived behavioral control	0.690^∗∗^	0.180^∗∗^	0.746^∗∗^	1		
Past behavior	0.703^∗∗^	0.245^∗∗^	0.766^∗∗^	0.739^∗∗^	1	
Perceived benefits	0.683^∗∗^	0.278^∗∗^	0.737^∗∗^	0.803^∗∗^	0.740^∗∗^	1

^∗∗^Correlation is significant at the 0.01 level (2-tailed).

**Table 4:** Healthcare students' and professionals' responses to intention items on raising public awareness of antimicrobial resistance (*N* = 572).

Statement	Intention to do (*N* = 572)	
	Yes *N* (%)	No/uncertain *N* (%)
1. I intent to address the public enquiries with accurate up-to-date information about the antimicrobial resistance	403 (70.5)	169 (29.5)
2. I am planning to educate the community about personal and environment hygiene	**421 (73.6)**	**151 (26.4)**
3. I want to use the social media to raise the public awareness about the antimicrobial's use and misuse	396 (69.2)	176 (30.8)
4. I am planning to broadcasting on TV channel and national newspapers to sensitize society about antimicrobial resistance and rationalizing antibiotic use	**340 (59.4)**	**232 (40.6)**
5. I am willing to collaborate with other healthcare professionals for infection prevention and control	**421 (73.6)**	**151 (26.4)**
6. I intent to reduce or stop the use of antibiotics in animals for human consumption because it is an important cause of appearance of new resistance to pathogenic agents	383 (67)	189 (33)
Overall intention to raise the public awareness and understanding of antimicrobial resistance	**425 (74.3)**	**147 (25.7)**

*Note:* Bold values indicate the highest, lowest, and overall percentages in each column for ease of comparison.

**Table 5:** Binary logistic regression of the predictors of healthcare students' and professionals' intention towards raising public awareness of antimicrobial resistance (*N* = 572).

Factor	Intention towards raising public awareness of antimicrobial resistance		COR	*p* value	95% CI for COR	AOR	*p* value	95% CI for AOR
	Yes (*N* = 425) # (%)	No/uncertain (*N* = 147) # (%)						
Gender								
Male	244 (71.8)	96 (28.2)	0.716	0.094	0.485–1.058	0.755	0.402	0.390–1.46
Female (ref)	181 (78.0)	51 (22.0)						
Age in years				0.007^∗^			**0.446**	
18–24	128 (65.6)	67 (34.4)	0.491	0.017^∗^	0.273–0.880	0.404	0.181	0.107–1.52
25–30	111 (76.6)	34 (23.4)	0.838	0.585	0.445–1.580	0.776	0.669	0.242–2.48
31–40	112 (80.6)	27 (19.4)	1.065	0.851	0.553–2.053	0.776	0.636	0.271–2.22
More than 41 (ref)	74 (79.6)	19 (20.4)						
Years of experience				0.043^∗^			**0.691**	
Undergraduate student	121 (72.0)	47 (28.0)	0.492	0.025^∗^	0.265–0.913	0.531	0.326	0.150–1.88
< 5 years	121 (69.1)	54 (30.9)	0.428	0.006^∗^	0.233–0.788	0.513	0.245	0.166–1.58
5–10 years	94 (76.4)	29 (23.6)	0.619	0.158	0.318–1.204	0.725	0.556	0.249–2.11
> 10 years (ref)	89 (84.0)	17 (16)						
Education level				**0.130**			**0.934**	
Undergraduate student	108 (70.6)	45 (29.4)	0.460	0.055	0.208–1.016	0.677	0.603	0.156–2.94
Bachelor	228 (73.1)	84 (26.9)	0.520	0.090	0.244–1.107	0.670	0.544	0.183–2.45
Master	42 (82.4)	9 (17.6)	0.894	0.828	0.324–2.462	0.612	0.549	0.123–3.05
PhD (ref)	47 (83.9)	9 (16.1)						
Field of education				**0.112**			**0.051**	
Pharmacy	177 (78.0)	50 (22.0.)	0.885	0.834	0.283–2.766	0.510	0.405	0.104–2.50
Medicine	68 (74.7)	23 (25.3)	0.739	0.620	0.224–2.44	0.275	0.137	0.050–1.51
Nursing	81 (64.8)	44 (35.2)	0.460	0.188	0.145–1.46	0.165	0.029	0.033–0.832
Medical laboratory sciences	51 (79.7)	13 (20.3)	0.981	0.976	0.280–3.44	0.302	0.173	0.054–1.69
Physiotherapy	32 (71.1)	13 (28.9)	0.615	0.454	0.173–2.19	0.344	0.246	0.057–2.084
Dentistry (ref)	16 (80.0)	4 (20.0)						
Marital status				**0.101**			**0.759**	
Single	205 (70.7)	85 (29.3)	1.378	0.616	0.393–4.83	2.934	0.350	0.307–28.02
Married	187 (77.6)	54 (22.4)	1.979	0.290	0.558–7.01	2.355	0.435	0.274–20.20
Divorced	26 (86.7)	4 (13.3)	3.714	0.112	0.737–18.7	1.628	0.696	0.141–18.83
Widow (ref)	7 (63.6)	4 (36.4)						
Attitude								
Positive	134 (82.2)	29 (17.8)	1.872	0.007^∗^	1.19–2.94	1.416	**0.295**	0.738–2.73
Negative/neutral (ref)	291 (71.1)	118 (28.9)						
Subjective norm	Median = 12 IQR = 11-14	Median = 6 IQR = 4-9	1.795	<0.001^∗^	1.63–1.97	1.823	<0.001^∗^	1.65–2.02

*Note:* Ref = reference category. Bold values denote the overall p values for each variable.

Abbreviations: AOR = adjusted odds ratio, COR = crude odds ratio, IQR = interquartile range.

^∗^ *p* value < 0.05.

## Data Availability

The authors confirm that the core data supporting the findings of this study are available within the article. Anonymized summary data and relevant analysis outputs are available upon request for research purposes. Full participant data are protected due to privacy regulations.
