# Correction to “Assessment of Healthcare Professionals’ and Students’ Perspectives and Intentions for Raising Public Awareness and Comprehension of Antimicrobial Resistance”

**DOI:** 10.1155/cjid/9785695

**Published:** 2026-06-16

M. S. Mohamed, E. A. A. Elhag, A. Ibrahim, et al., “Assessment of Healthcare Professionals’ and Students’ Perspectives and Intentions for Raising Public Awareness and Comprehension of Antimicrobial Resistance,” *Canadian Journal of Infectious Diseases and Medical Microbiology* 2025 (2025): 4106594, https://doi.org/10.1155/cjid/4106594.

In the article titled “Assessment of Healthcare Professionals’ and Students’ Perspectives and Intentions for Raising Public Awareness and Comprehension of Antimicrobial Resistance”, author Mona Timan Idriss was not affiliated to Department of Medical Sciences and Preparation Year, Northern College of Nursing, Arar 73312, Saudi Arabia which is incorrect. The correct affiliations for this author are:

Nursing Department, North Private College of Nursing, Arar, Saudi Arabia.

The corrected list of affiliations is shown in the author information above.

We apologize for this error.

